# Neuropsychiatric Symptoms in Parkinson’s Disease: Beyond Complications

**DOI:** 10.3389/fpsyt.2016.00110

**Published:** 2016-06-23

**Authors:** Angie A. Kehagia

**Affiliations:** ^1^Institute of Psychiatry, Psychology and Neuroscience, King’s College London, London, UK

**Keywords:** Parkinson’s disease, symptoms, complications, nosology, neuropsychiatry

Parkinson’s disease (PD) extends beyond movement to often encompass a range of psychiatric phenomena, such that it has been viewed as the prototypical neuropsychiatric disorder (1). While the movement disorder is primarily linked to a central dopamine deficit, neurodegenerative events in the main noradrenergic, serotoninergic, and cholinergic nuclei precede motor symptom onset, by a decade or longer (2, 3). Thus, the high prevalence of neuropsychiatric symptoms could be seen as the inexorable consequence of a multifaceted neuropathology, and some of these are, in fact, recommended by the EFNS/MDS-ES Task force as criteria to aid differential diagnosis (4).

Subtle tensions regarding the ontology of psychiatric phenomena in PD are nonetheless apparent in that they are variously referred to as symptoms, linking them directly to the disease, complications arising from treatments that target dopamine to alleviate motor symptoms, or more generally as dysfunction or impairment. This reluctant consensus most likely mirrors the evolving state of the science on the complex problem of resolving symptoms from complications in PD, which poses a number of challenges. Disabling motor impairment rules out the possibility of withdrawing medication in order to reveal the psychiatric sequelae of the disease unconfounded by it. Moreover, these behaviors may not be seen in newly diagnosed, drug naive patients due to their time course and the inherent heterogeneity of the disease. However, resolving the nosological status of psychiatric symptoms in a movement disorder, which fall between the remits of neurology and psychiatry, in an evidence-based manner and systematically integrating them into the framework of a disease of the brain with both motor and psychiatric manifestations arising from pervasive degenerative change is important. Delineating symptoms and differentiating them from complications in PD has clear implications for patients’ perceptions of their illness, their attributions, choices, and communication with clinicians, and, ultimately, effective clinical management.

Early psychiatric changes in people who go on to develop motor symptoms consistent with PD represent compelling candidates for the category of neuropsychiatric symptoms that stem from the disease itself.

Anxiety disorders (5, 6) and depression (7) may precede the onset of motor symptoms by one or even two decades and significantly increase the risk of a future PD diagnosis. In addition, some patients who experience on–off fluctuations in motor function also experience profound fluctuation in mood, which can rapidly change from depressed and suicidal to euphoric or even hypomanic (8). Mood swings are not reliably linked to dopaminergic dosing or motor symptom deterioration (9, 10), but are rather seen more often in patients with a pre-existing psychiatric history or concurrent use of psychiatric medication (11), suggesting these may in fact be part of a larger symptom constellation. Thus, while the general population prevalence of depression suggests that at least in some people with PD this will be comorbid and psychological and psychosocial factors may also be at play, convergent evidence indicates that for many others this will be accurately, and usefully, conceptualized as a symptom of PD. For some patients, mood disturbance predates motor symptoms and coincides chronologically with the degenerative changes in the locus coeruleus and raphe nuclei seen in the earliest Braak stages leading to early compromise on monoaminergic processes. Depression is the second highest quality-of-life predictor in PD (12), and, notably, patients who invoke PD as a major cause for their symptoms attribute these to perceived psychosocial effects related to their illness rather than its neurobiology (13). Such attributions and an emphasis on motor symptoms in patient reports and clinician investigations which often overlook non-motor symptoms (14) may contribute to their underdiagnosis and undertreatment (15).

REM sleep behavior disorder (RBD) is another prodromal symptom of PD indicative of a synucleinopathy, where noradrenergic deficits in particular, arising due to the degeneration of the locus coeruleus, appear to play an important role (16).

Conversely, symptoms also emerge in the course of the heterogeneous progression of PD. While dementia appears in some patients at the end of a long disease course, in others, this profound cognitive decline occurs relatively early on. The early features of cognitive decline leading to dementia, namely, early visuospatial and memory deficits associated with posterior cortical compromise and evidence of cholinergic dysfunction (17), appear to be distinct from the more prevalent pattern of mild cognitive impairment in the form of executive deficits that mirror monoaminergic dysfunction (18). Thus, profound cholinergic deficits, amyloid and synuclein aggregates contribute to dementia [for review, see Ref. (19)] and demarcate it as a distinct neuropsychiatric symptom within the heterogeneity of cognitive impairment seen in PD.

Other psychiatric changes remain less well understood as symptoms arising from the disease or complications due to medication or a combination of both. Yet, some inferences are possible. For example, episodes of delirium in PD can be linked to anticholinergic medication, in which case these can be considered an iatrogenic complication. However, in some patients, delirium is a symptom mirroring neurotransmitter dysregulation and linked, in particular, with serum anticholinergic activity (20). Although the cholinergic, dopaminergic as well as inflammatory changes [for review, see Ref. (21)], which are thought to underlie delirium in PD, are ill-specified, distinguishing an event that might resolve quickly once a drug is removed from a recurrent pattern indicative of an underlying disease process is important in informing clinical management. This is a particularly salient consideration in the case of delirium episodes, the ensuing hysteresis in recovery to previous levels of functioning, and the robust association with accelerated cognitive decline and mortality in the elderly (22).

Moreover, although PD psychosis, a constellation of hallucinations, delusions, and other symptoms such as sense of presence (23), used to be attributed to dopaminergic overstimulation by ergot DA agonists in particular (24), its broad phenomenology and the lack of a conclusive relationship to levodopa or levodopa-equivalent dose (LED) in cross-sectional studies [for review, see Ref. (25)] suggest a much more complex, if ill-defined, picture. While for some patients, abnormal experiences such as visual hallucinations may be short-lived if the dose of dopaminergic medication can be reduced, for other patients, psychosis may herald or coincide with dementia and is the highest predictor of nursing home placement (26). Thus, PD psychosis is currently understood as emerging from the interaction of an idiosyncratic disease process with long-term exposure to dopaminergic medications: greater age at onset, motor symptom severity dictating higher dopaminergic dosing, cognitive impairment, and REM sleep behavior disturbance (27) reliably increase the risk for PD psychosis (28), indicating that parallel, non-dopaminergic, disease-related pathologies are additionally at play. As these remain poorly understood, they cannot translate into targeted pharmacological intervention. For example, atypical antipsychotics such as clozapine and quetiapine with dual dopaminergic and serotonergic action are among the most commonly used in the management of psychosis in PD, with comparable efficacy in head-to-head trials (29). However, the use of quetiapine is not well supported by clinical trials but appears to proceed largely on the basis of favorable clinician impressions (30, 31). This disparity raises questions that may be addressed in the context of large-scale trials employing stratified designs and multivariate approaches, equipped to negotiate the heterogeneity of the disease and nature of these symptoms. Furthermore, the success of the selective 5HT2A inverse agonist pimavanserin in reducing the positive, but not all the symptoms of psychosis (32), suggests that some of its aspects are at minimum amenable to serotonergic leverage, although a direct causal role of a serotonergic imbalance has yet to be established.

The psychiatric phenomena that may be currently said to stand as clear treatment complications are impulse control disorders (ICDs), collectively referring to gambling, hypersexuality, punding, as well as dopamine dysregulation syndrome (DDS), the compulsive use of antiparkinsonian drugs, which can lead to drug induced on-period hypomania (33). DA agonists in particular (34, 35) have been directly implicated in triggering ICDs in vulnerable patients, typically younger, male, and with distinctive ventral corticostriatal dopaminergic pathophysiology (36). Given that DA agonists can produce ICDs in patients without PD (restless leg syndrome and pituitary adenoma), these behaviors are accurately conceptualized as iatrogenic complications.

Psychiatric complications are sometimes also seen after deep brain stimulation in a subset of patients and can include ICDs, mania, and depression, especially when stimulation targets the subthalamic nucleus (37–39). These phenomena are complications by definition, but their management still represents a challenge, particularly in the case of complications such as suicidality, where the identification of risk factors and intervention in the case of preventable ones is a priority (40).

Collectively, these observations support a nosological argument for distinguishing *bona fide* neuropsychiatric symptoms of PD from complications, predicated on acknowledging the need for a better understanding of its heterogeneous process. Certain psychiatric symptoms may precede the PD diagnosis itself, representing identifiable and clinically relevant disease prodromes linked directly to different aspects of neuropathology. The fact that severe mood disturbance and RBD represent prospective diagnostic criteria for PD constitutes clear evidence that the concept of the disease is evolving beyond striatal dopamine and motor dysfunction. Other psychiatric manifestations, such as episodes of delirium and psychosis, appear as PD progresses and may be exacerbated by medication. Their persistence despite the removal of potential pharmacological culprits where possible points to a distinctive underlying disease profile, which combined biomarker approaches may eventually isolate, and thus lend support to their classification as symptoms. ICDs are better understood to represent a complication of treatment with a specific drug class, now used with caution, as are post-operative psychiatric complications following DBS. Beyond better science, the profound impact of psychiatric symptoms on the quality of life of people living with PD, as well as its underdiagnosis, represents the most compelling observation that multidisciplinary approaches that transcend Cartesian dualism and the borders of medical specialisms is also a necessity.

## Author Contributions

AAK is the sole contributor of this work and has approved it for publication.

## Conflict of Interest Statement

The author declares that the research was conducted in the absence of any commercial or financial relationships that could be construed as a potential conflict of interest.
